# Systemic sclerosis complicated with renal thrombotic microangiopathy: a case report and literature review

**DOI:** 10.1186/s12882-021-02639-w

**Published:** 2022-01-10

**Authors:** Weiwei Kong, Yaomin Wang, Huiping Wang, Qin Zhou, Jianghua Chen, Fei Han

**Affiliations:** Kidney Disease Center, the First Affiliated Hospital, Zhejiang University School of Medicine; Key Laboratory of Kidney Disease Prevention and Control Technology, Zhejiang Province; National Key Clinical Department of Kidney Diseases; Institute of Nephrology, Zhejiang University; Zhejiang Clinical Research Center of Kidney and Urinary System Disease, No.79, Qingchun Road, Shangcheng District, Hangzhou, Zhejiang China

**Keywords:** Overlap syndrome, Scleroderma renal crisis, Systemic sclerosis, Thrombotic microangiopathy, Case report

## Abstract

**Background:**

Systemic sclerosis (SSc) may overlap with other connective tissue diseases, which is named overlap syndrome. Scleroderma renal crisis (SRC) is a rare but severe complication of SSc. SSc related thrombotic microangiopathy (SSc-TMA) is an infrequent pathology type of SRC, while SSc-TMA accompanied by overlap syndrome is very rare.

**Case presentation:**

This study reported a case of acute kidney injury (AKI) accompanied with overlap syndrome of SSc, systemic lupus erythematosus (SLE) and polymyositis (PM). The renal pathology supported the diagnosis of SSc-TMA but not SLE or PM-related renal injury, characterized by renal arteriolar thrombosis, endothelial cells edema, little cast in tubules and mild immune complex deposition. The primary TMA related factors (ADAMTS13 and complement H factor) were normal. Thus, this case was diagnosed as secondary TMA associated with SSc. The patient was treated with renin angiotensin system inhibitors, sildenafil, supportive plasma exchange/dialysis, and rituximab combined with glucocorticoids. After 2 months of peritoneal dialysis treatment, her renal function recovered and dialysis was stopped.

**Conclusion:**

This study presented a case of SSc-TMA with overlap syndrome. Rituximab can be used as a treatment option in patients with high SRC risk or already manifesting SRC.

## Background

Overlap syndrome is defined as an entity that satisfies the classification criteria of at least two connective tissue diseases (CTDs) occurring at the same time or at different times in the same patient. CTDs include systemic lupus erythematosus (SLE), rheumatoid arthritis (RA), systemic sclerosis (SSc), polymyositis/dermatomyositis (PM/DM), and Sjögren syndrome (SS). CTDs can overlap in various ways. The most observed overlap syndrome is the combination of two CTDs. However, the combination of three different CTDs, such as SLE, SSC, and PM in our case, was rarely reported [1].

Scleroderma renal crisis (SRC) is a rare (seen in 5 to 10% of SSc patients) but severe complication of SSc. According to histopathological changes, SRC can be divided into 2 types, narrowly defined SRC (nd-SRC) and SSc-associated thrombotic microangiopathy (TMA). nd-SRC is a typical type of SRC, which shows acute renal failure and abrupt onset of moderate-to-significant hypertension. The pathology of nd-SRC shows injured endothelial cells and subsequent intimal thickening in the arcuate and interlobular arteries. SSc-TMA is a rare type of SRC, with increased serum creatinine, thrombocytopenia, hemolysis, and proteinuria or hematuria. The pathology of SSc-TMA shows abnormalities in the wall of arterioles and capillaries which eventually leads to microvascular thrombosis [2–4].

The occurrence of SSc-TMA is infrequent and SSc-TMA accompanied with overlap syndrome is even rare. Here we reported a case of SSc-TMA in a patient with overlap syndrome of SSc, SLE and PM. Informed written consent was obtained from the patient for the publication of this case report and its accompanying images.

## Case presentation

A 21-year-old female patient was first admitted for high fever, swelling and pain in the lower limbs. Physical examination revealed that she had swollen knee joints, stiff skin, Raynaud’s phenomenon in the fingertips, and normal blood pressure. Routine blood examination showed white blood cell count of 3.7 × 10^9^/L with 70.3% neutrophilic granulocyte and hemoglobin 102 g/L. Her C-reactive protein was 17.2 mg/L. Her antinuclear antibodies (ANA) test was positive, with positive antibodies of anti-Scl-70, anti-SSA, and anti-Ro-52. She also had low complement and normal serum creatinine of 68 μmol/L. Her chest computerized tomography (CT) showed infection in the lobe of the right middle lung. She was administered antibiotics, immunoglobulin, methylprednisolone 40 mg/day, and rituximab 100 mg twice (interval period of 1 week). After treatment, her symptoms were relieved and she was discharged. Two weeks after being discharged, she complained of gross hematuria accompanied by nausea, vomiting**,** backache, and decreasing urine volume. Then she was re-admitted.

Physical examination at her second admission revealed that she had swollen knee joints, stiff skin, and mild symmetrical edema in the lower limbs. No weakness of the muscles. The pulmonary and cardiac tests showed no pathological findings. The blood pressure was 141/101 mmHg.

Her blood routine analysis showed a hemoglobin level of 42 g/L, platelet count of 24*10^9^/L, and reticulocyte of 20.68%. Her biochemical analysis showed that her serum creatinine was 118 μmol/L, which rapidly increased to 424 μmol/L in 2 days. She had normal aminotransferases, creatine kinas 353 U/L, creatine kinas-MB 57 U/L, total bilirubin 84.7umol/L, indirect bilirubin 65.3umol/L, and lactic dehydrogenase 1712 U/L. Her urinalysis showed albumin +, red blood cells 20/ul, occult blood +++, proteinuria creatinine ratio 86.83 mg/g. She had normal C-reactive protein but high erythrocyte sedimentation rate of 55 mm/h, low complement 4 (C4) 0.04 g/L and complement 3 (C3) 0.28 g/L, low plasma complements H factor (CHF) concentration 135.63 ng/ml (reference range 210–452.5 ng/ml), and negative anti-CHF antibody. She had positive ANA 1:1000 with anti-Scl-70 +, anti-SSA +, anti-Ro-52 +, and anti-dsDNA-. She had normal activity of ADAMTS13 (74.2%, reference range 68–131%), negative anti-ADAMTS13 antibody, positive Coomb’s test, and 6–11% fragmented red blood cells in peripheral blood.

Her pulmonary CT showed a small-sized pericardial effusion without obvious pulmonary interstitial change. Her heart echo showed pulmonary hypertension and her urinary ultrasound revealed decreased blood flow signal in both kidneys.

**Fig. 1:**
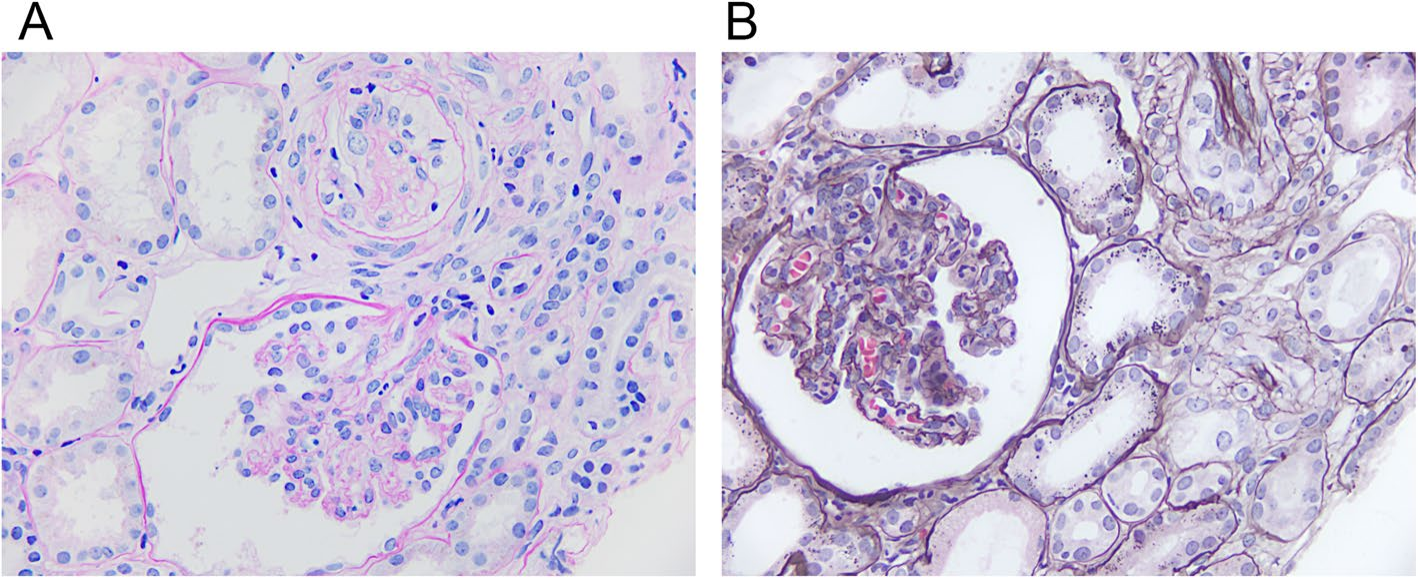
Stenosis and ischemia in glomerular capillary loops; moderately thickened vessels; edema endothelial, intimal thickening and occlusion in arteries (**A**, PAS; **B**, PASM; Magnification, × 400)

Renal, skin, and muscle biopsies were performed after her recovery from anemia and thrombocytopenia. Her skin biopsy showed reticulodermal keratosis, local basal cell proliferation, tpigmentation of the basal layer, disappearance of epidermal protrusion, proliferation of small blood vessels, and proliferation of collagen fiber in the superficial dermis. The above data met with pathology changes of scleroderma skin. Her muscle biopsy showed the pathology change of inflammatory myopathy. Her renal biopsy showed TMA changes in the kidney. The walls of the renal arterioles were thickened due to endothelial edema and endometrial fibrosis. The renal arterioles’ lumen was diffusely narrow and occluded. The glomerulus capillary loops were diffusely narrow and the glomerulus showed diffused ischemia. Electron microscopy showed occlusion of the vascular lumen, widening of the basement membrane, and the diffused fusion of the foot process (Fig. 1).

Her diagnoses were overlap syndrome of SSc, SLE and PM, and secondary TMA caused AKI. She was treated with glucocorticoids (methylprednisolone 500 mg/day for 3 days, then prednisone with gradually tapered dose), rituximab 500 mg twice with 2 weeks’ interval, renin-angiotensin system inhibitors, and sildenafil. Also, she was administered emergency hemodialysis (HD) and plasma exchange (PE) for 9 times. After the above treatment, her anemia and thrombocytopenia were quickly recovered. Her sedimentation rate and complement became normal. However, her renal function did not recover and she was switched to peritoneal dialysis (PD) before discharge. At discharge, we added mycophenolate mofetil (250 mg twice per day) and hydroxychloroquine (200 mg twice per day) to maintain the immunosuppressive treatment. Two months after being discharged, her serum creatinine decreased to 292 μmol/L and her urine volume increased to 1200 ml per day. Then, she stopped peritoneal dialysis. Her serum creatinine decreased to 109 μmol/L 8 months after her first admission.

## Discussion and conclusions

Overlap syndrome is an infrequent systemic autoimmune disease with an overlapping feature of at least two CTDs [1]. In the current case, the patient was diagnosed with an overlap syndrome of SSc, SLE, and PM based on the corresponding classification criteria. The patient exhibited sclerodactyly and Raynaud’s phenomenon during her first admission accompanied by the positive anti-Scl-70 antibody. According to the 2013 European League Against Rheumatism (EULAR)/American College of Rheumatology (ACR) classification criteria for SSc, the patient got a total score of 10 and can be diagnosed as SSc [5]. Her skin biopsy also supported the diagnosis of SSc. In addition, the patient presented fever, leukopenia, hemolysis, pericardial effusion, musculoskeletal joint involvement, low C3, and low C4. Based on the 2019 EULAR/ACR classification criteria for SLE, the patient got a total score of 21 and reached the criteria for SLE [6]. The diagnosis of PM mainly depended on elevated serum levels of creatine kinase, and perifascicular atrophy in muscle biopsy. The patient was graded based on the 2017 EULAR/ACR classification criteria for idiopathic inflammatory myopathies (IIM) and got a score of 9.2 (8.7 is the cut-off line for diagnosis) [7].

AKI occurred during the treatment period after the diagnosis of the overlap syndrome. All three kinds of primary autoimmunity diseases, PM, SSc, and SLE, can induce AKI. SSc-induced AKI normally presents SRC [8]. The primary pathogenic process is thought to be an injury to the endothelial cells resulting in intimal thickening and proliferation of the renal intralobular and arcuate arteries. The renal biological data in our case supported the diagnosis of SRC and presented as thickened renal arterioles, thrombosis, edema in endothelial cells, fibrous proliferation, and glomerular ischemic changes. SLE is characterized by the dysregulation of the immune system which consequently results in the formation of immune complex. The immune complex results in the injury to the endothelial cells and the recruitment of immune cells, which is the key mechanism in the development of lupus nephritis [9]. However, in this case, the renal biopsy showed that glomerular immune complex deposition was not obvious and only mild IgM deposition in mesangial areas. Furthermore, no proliferative or membranous changes were observed under light microscope. Thus, based on the renal pathological data, lupus nephritis was not considered. Rhabdomyolysis is a complication of PM, which may lead to acute tubular necrosis with deterioration of renal function [10]. Both clinical tests and renal biopsy of our case did not support the occurrence of rhabdomyolysis, because no extra-high creatine kinase, myoglobinuria, or tubular casts were shown.

The renal pathology in our case was characterized by TMA change. TMA can be observed in a wide spectrum of clinical scenarios, which includes but is not limited to thrombotic thrombocytopenic purpura (TTP), atypical hemolytic uremic syndrome (aHUS), antiphospholipid antibody syndrome, scleroderma renal crisis, drug toxicities, or metabolic disorders [11]. Depending on the causes of TMA, TMA classification includes primary TMA, secondary TMA, and infection-associated TMA [12]. TTP and aHUS are two common types of primary TMA. TTP is associated with a severe deficiency of ADAMTS13. The deficiency of ADAMTS13 leads to the formation of platelet-rich thrombi in the microvasculature. The severe deficiency of ADAMTS13 appearing in TTP is defined as < 5–10% of normal protease activity. ADAMTS13 activity > 10% can help in the exclusion of TTP diagnosis [13–15]. Our case showed normal ADAMTS13 activity (74.2%) and negative ADAMTS13 antibody. Defects in CFH are the common reason for aHUS, which can lead to failure of C3 breakdown and activation of terminal complement pathway. It has been reported that 60% less than normal level of CFH is regarded as a quantitative deficiency in aHUS [14, 16]. The level of CFH concentration in our case was lower than normal reference range but with no meaningful defects, which did not support the diagnosis of aHUS. SLE and SSc, two primary diseases in our case are associated with secondary TMA. Besides classical TMA change, the TMA secondary to SLE and SSc has its pathological characteristics. The TMA secondary to SSc shows the characteristics of vascular endothelial injuries and proliferation on small arterioles [12]. The SLE-induced TMA shows immune complex deposition and complementary activation in the kidney [17]. The renal biopsy data in our case showed obvious vascular endothelial edema and proliferation, but little immune complex or complementary deposition. Thus, we inferred that the TMA changes in this case were secondary to SSc but not SLE.

Glucocorticoids are the first-line treatment for SSc/SLE/PM overlap syndrome. But the treatment of glucocorticoids in SSc is considered a significant risk factor for SRC irrespective of whether it is medium, high, or prolonged use of low doses [18]. Rituximab (RTX) is an antibody that targets CD20 and treatment with RTX leads to the depletion of B lymph cells [19]. RTX has been considered as a treatment option for SSc to avoid increasing the dosage of glucocorticoids and thus decrease the risk for SRC. In Balazs Odler1’s research, patients responded well to the combination treatment with prednisolone and RTX (RTX 500 mg on day 0 and day 14, then administered twice every 3 months). No SRC or significant drop in kidney function was observed during an average follow-up period of 3.4 years [20]. For patients who already get SRC, RTX combined with glucocorticoids can also achieve good response. A recent case reported that a 56-year-old woman with overlap syndrome of SSc and PM developed into SRC. The combination of glucocorticoids and RTX resulted in successful remission [21]. Similarly, glucocorticoids and RTX treatment in our case resulted in good response. We indicated that rituximab could be considered as a treatment option in patients who have high risk for SRC or already with SRC in overlap syndrome. But long-term outcomes after remission remained to be further established.

Our patient showed slow recovery of renal function. According to previous reports, AKI induced by SSc-TMA potentially has a likelihood for the recovery of endogenous renal function [22]. Because PD provides better preservation of residual kidney function (RKF) compared to HD, PD was used in the prolonged AKI period in our case. The hemodynamic status during dialysis in PD is more stable than HD. In addition, the membrane used in hemodialyzers is less biocompatible than peritoneal membrane, leading to RKF loss caused by repeated exposure to inflammatory mediators generated by the extracorporeal circulation [23]. After 2 months of PD, this patient gradually attained the recovery of her renal function and stopped dialysis.

To the best of our knowledge, only five cases of overlap syndrome accompanied with SSc-TMA have been reported in English (Table 1). They showed different prognosis. Two reported cases (case 3 [24] and case 5 [25]) became HD dependent during follow up, two cases (case 1 [26] and case 4 [27]) died because of severe complications of diffused alveolar hemorrhage and heart failure, and one case (case 2 [28]) showed recovery to normal kidney function. The case with good prognosis presented acute tubular necrosis and mild TMA change in renal biopsy. Two dialysis-dependent cases showed severe occluded vascular lumen and even mesangiolysis.

**Table 1:** Summary of reported SCC-TMA cases in overlap syndrome

	Current case	Case 1	Case 2	Case 3	Case 4	Case 5
Author	–	Hongrui Dong	Jordana Cheta	Greenberg	Yuki Nanke	Carlos Quereda
Year	–	2020	2017	2001	2000	1991
Age (year)	21	61	50	64	33	47
Gender	Female	Male	Female	Female	Female	Female
CTD	SCC-SLE-PM	SCC-SLE	MCTD	SCC-DM	SCC-SLE-RA	SCC-PM
CTD History	0y	5y	10y	0y	7y	2y
Other organs	–	CHF ILD	–	–	DAH	ILD
Renal pathology	Stenosis and ischemia in glomerular capillary loops; moderately thickened vessels; edema endothelial, intimal thickening and occlusion in arteries.	Mucinous edema and “onion bulbs” in interlobular arteries; widened loose layer of glomerular basement membrane.	1/9 glomerulus had a thrombus within an afferent arteriole; moderately thickened vessels; multiple red cell casts and dilated tubules.	Severe thrombotic and mesangiolysis in glomerular; multiple sclerosis or occluded vascular lumen with thrombi; focal tubular necrosis.	Multiple thrombi in afferent arterioles of glomeruli; fibrous and edematous arteries.	Multiple thrombi in glomerular capillary loops; endothelial proliferation, intimal thickening and thrombi occlusion in arteries.
Treatment	CS, RASI, HD-PD, RTX	CS, CTX, RASI	CS, RASI, HD	CS, RASI, HD	CS, CTX, RASI HD, PE	CTX, RASI, HD-PD
Prognosis	Normal renal function	Death	Normal renal function	Dependent on dialysis	Death	Dependent on dialysis

Note: *CHF* congestive heart failure; *CS* corticosteroid; *CTD* connective tissue disease; *CTX* cyclophosphamide; *DAH* diffuse alveolar hemorrhage; *DM* dermatomyositis; *HD* hemodialysis; *ILD* interstitial lung disease; *MTCD* mixed connective tissue disease; *PD* peritoneal dialysis; *PE* plasma exchange; *PM* polymyositis; *RA* rheumatoid arthritis; *RASI* renin-angiotensin-aldosterone system inhibitor; *RTX* rituximab; *SCC* systemic sclerosis; *SLE* systemic lupus erythematosus; *y* year

In summary, we reported a rare case of SSc-TMA with overlap syndrome (SCC/SLE/PM). This report provides evidence that renal biopsy and immunology test help to differ causes of AKI in overlap syndrome, make reasonable treatment plans, and effectively predict the prognosis. Furthermore, rituximab can be considered a treatment option in patients who have a high risk for SRC or already presented SRC in overlap syndrome.

## Data Availability

All data in the study are included in this published article.

## Abbreviations

ADAMTS13: A disintegrin and metalloproteinase with a thrombospondin type 1 motif, member 13
AKI: Acute kidney injury
ACR: American College of Rheumatology
aHUS: Atypical hemolytic uremic syndrome
C4: Complement 4
C3: Complement 3
CHF: Complement H factor
CT: Computerized tomography
CTDs: Connective tissue diseases
DM: Dermatomyositis
EULAR: European League Against Rheumatism
HD: Hemodialysis
IIM: Idiopathic inflammatory myopathies
OS: Overlap syndrome
PD: Peritoneal dialysis
PE: Plasma exchange
PM: Polymyositis
RA: Rheumatoid arthritis
RKF: Residual kidney function
RTX: Rituximab
SRC: Scleroderma renal crisis
SLE: Systemic lupus erythematosus
SSc: Systemic sclerosis
TTP: Thrombotic thrombocytopenic purpura
TMA: Thrombotic microangiopathy
